# Presence of High-Risk HPV mRNA in Relation to Future High-Grade Lesions among High-Risk HPV DNA Positive Women with Minor Cytological Abnormalities

**DOI:** 10.1371/journal.pone.0124460

**Published:** 2015-04-20

**Authors:** Hanna Johansson, Kaj Bjelkenkrantz, Lotten Darlin, Joakim Dilllner, Ola Forslund

**Affiliations:** 1 Department of Laboratory Medicine, Medical Microbiology, Lund University, Malmö, Sweden; 2 Regional Cancer Centre South, Lund, Sweden; 3 Department of Obstetrics and Gynecology, Skane University Hospital, Lund, Sweden; 4 Department of Medical Epidemiology and Biostatistics, Karolinska Institutet, Stockholm, Sweden and Department of Laboratory Medicine, Karolinska Institutet, Stockholm, Sweden; Georgetown University, UNITED STATES

## Abstract

**Objective:**

Continuous expression of E6- and E7-oncogenes of high-risk human papillomavirus (HPV) types is necessary for the development and maintenance of the dysplastic phenotype. The aim of the study was to determine the sensitivity and specificity of the APTIMA HPV mRNA assay (Hologic) in predicting future development of high-grade cervical intraepithelial neoplasia (CIN) among high-risk HPV-DNA-positive women with atypical squamous cells of undetermined significance (ASCUS) or low-grade squamous epithelial lesion (LSIL) cytology.

**Methods:**

Archived SurePath cervical samples of women ≥ 35 years of age with high-risk HPV DNA-positive ASCUS (n = 211) or LSIL, (n = 131) were tested for the presence of high-risk HPV E6/E7 mRNA using the APTIMA HPV assay, and the women were monitored for development of histopathologically verified CIN2+.

**Results:**

Twenty-nine percent (61/211) of the women in the ASCUS group, and 34.3% (45/131) in the LSIL group developed CIN2+ within 4.5 years of follow-up. The prevalence of HPV mRNA was 90.0% (95% CI 85.9-94.0) among women with ASCUS and 95.4% (95% CI 91.8-99.0) among women with LSIL. The presence of HPV E6/E7 mRNA was associated with future development of CIN2+ among women with ASCUS and LSIL (p=0.02). The mRNA assay demonstrated high sensitivity in predicting future CIN2+ and CIN3 for index ASCUS (96.7%; 95% CI 87.6-99.4 and 100%; 95% CI 82.2-100, respectively) and LSIL (97.8%, 95% CI 86.8-99.9 and 100%, 95% CI 79.9-100, respectively). The corresponding specificity was low, 12.7% (95% CI 7.9-19.3) and 5.8% (95% CI 2.2-13.6), for future CIN2+, respectively. The negative predictive value of the HPV mRNA assay for detecting future CIN3 was 100%, since no mRNA-negative woman developed CIN3 (0/27) as compared to 13.6% (43/315) of the mRNA-positive women (p = 0.03).

**Conclusion:**

The APTIMA mRNA assay demonstrated high sensitivity but low specificity in predicting future CIN2+ among women with minor cytological abnormalities. The assay had high negative predictive value for future CIN3, indicating that HPV-mRNA-negative women are at low risk of progression to high grade CIN.

## Introduction

Oncogenic human papillomaviruses (HPVs) are the main cause of cervical cancer, being found in close to 100% of cervical tumors [1]. The earliest manifestations of cervical lesions are low grade cytological abnormalities, i.e. atypical squamous cells of undetermined significance (ASCUS) and low-grade squamous epithelial lesions (LSIL). A systematic review of 423 studies showed that HPV-DNA of 48 different HPV types of the alpha papillomavirus genus was present in 52.1% of ASCUS and 74.2% of LSIL lesions [2]. A meta-analysis of 32 studies reported that high-risk HPV (HR-HPV) types are detectable in 43% (range 23–74%) of ASCUS and in 76% of CIN1 (range 55–89%) samples [3]. HR-HPV testing is recommended as triage of ASCUS [4, 5]. In southern Sweden, HPV reflex testing by the use of MGP-PCR and Luminex [6, 7] is used as triage for all women >35 years of age with ASCUS/LSIL cytology. Women with HR-HPV type(s) and ASCUS or LSIL are referred to colposcopy. However, for the vast majority (91%) of HPV DNA-positive women with ASCUS or LSIL the HPV infection will clear within about 6–24 months [8], and about 70% of ASCUS and low grade lesions regress spontaneously [9]. Continuous expression of HR-HPV E6 and E7 oncoproteins is necessary for transformation of normal cells to dysplastic cells [10]. Unnecessary colposcopies could be avoided if only those with relevant HPV infections, i.e. women with persistently active HPV infection, who are at risk of development of high-grade lesions, were referred. An assay that detects the mRNA of the HR-HPV E6/E7 oncogenes could have the potential to detect clinically relevant HPV infections [11, 12]. The aim of the study was to determine the sensitivity and specificity of the APTIMA HPV mRNA assay in predicting future development of high-grade cervical intraepithelial neoplasia among HR-HPV DNA-positive women with ASCUS or LSIL cytology.

## Patients and Methods

### Clinical samples

The screening population in the Malmö area of southern Sweden consists of approximately 175,000 women and about 34,000 cervical smears are taken annually. Within the Malmö area 15–20 new cases of invasive cervical cancer occur yearly, with an incidence of 10.7 per 100,000 women [13]. All liquid-based cytology (LBC) cervical samples collected between January of 2009 and December of 2010 were stored and entered into a database. The cervical samples were taken using SurePath (BD) and diagnosed according to the Bethesda system [14] at the Clinical Pathology and Cytology Department of Laboratory Medicine, Region Skåne in Malmö. For this study we have used ASCUS and LSIL LBC samples from women over and including 35 years of age that were pelleted and frozen at -80°C within a week after sampling. For routine HPV-DNA analysis, the pellets were thawed and re-suspended in 400 μL Specimen Transport Medium (STM) (Qiagen). The remaining material (200 μL) was re-frozen. At present, the HPV-DNA analysis, using MGP-PCR and Luminex detection of amplicons [6, 7], identifies 39 different HPV types; HPV6, 11, 16, 18, 26, 30, 31, 33, 35, 39, 40, 42, 43, 45, 51, 52, 53, 54, 56, 58, 59, 61, 62, 66, 67, 68, 69, 70, 73, 74, 81, 82, 83, 86, 87, 89, 90, 91 and 114.

We utilized the cytology and pathology registries of the Swedish Regional Cancer Centre South to identify index LBC samples of women aged 35 years or more, with cytological diagnoses of ASCUS or LSIL, and infected by at least one HR-HPV type (by HPV-DNA analysis).

The study population was composed of HR-HPV-positive women, all diagnosed with ASCUS (n = 211) or LSIL (n = 131). The median age of the women was 42 years for both the ASCUS (range 35–68 years) and the CIN1 (range 35–87 years) groups.

Registry data on histological follow-up were retrieved for each patient up to 4.5 years (end 2014-06-31) after the index cytology sample. The most severe histological diagnosis was recorded. The mean time for end-point diagnosis was 196 days (range 29–938). For the women without progression to CIN2+, data was last retrieved after a mean time of 249 days (range 17–1067).

The follow-up and treatment of the included women has not been influenced by the results of the HPV mRNA test. Of the women without progression to CIN2 or CIN3, 41.3% (62/150) of the non-progressing women with ASCUS at baseline were treated (conization; n = 58, hysterectomy; n = 4). Of the women with LSIL at baseline, 54.6% (47/86) were treated (conization; n = 45, hysterectomy; n = 2).

These women were not excluded as they had a chance to develop a more serious lesion than ASCUS and LSIL. Within this register-based study, women were followed until histopathological diagnosis of CIN2+, or until treatment that removes the cervix or until the end of the study at 2014-06-31, whichever came first.

### Ethical statement

At routine cervical cytology sampling patients gave consent to storage of their samples. For the current study no written or informed consent was obtained. Instead, information about the study was published in local newspapers, with an opt-out approach for participants to be excluded from the study through registration at the Biobank registry of the Lund University Hospital. Given that we performed a retrospective study that used registry data gathered by the Cytology and Pathology registries of the Swedish Regional Cancer Center South, the Ethical Committee waived the need for written informed consent. This study was approved by the Ethical Committee of Lund, Sweden (dnr 2013/185).

Linkage between the cytology-pathology registries and the LBC biobank of the Cytology Department of the Malmö Hospital was performed by the use of patients’ identification number by the hospital data manager (MH). Patient identities were de-identified before access to the researchers. We received anonymized medical data from HPV-DNA positive women over and including the age of 35, including age, date of initial ASCUS/LSIL diagnosis, time between sampling and freezing date at -80°C, HPV-DNA result, date and diagnosis of histological follow-up.

### Inclusion and exclusion criteria

Cervical LBC samples were included in the study if the sample were DNA-positive for any HPV type covered by the APTIMA HPV assay. The APTIMA HPV assay detects, but cannot distinguish between, E6/E7 mRNA of HPV16, 18, 31, 33, 35, 39, 45, 51, 52, 56, 58, 59, 66 and 68. The APTIMA HPV assay is known to cross-react with HPV types 26, 67, 70 and 82 (Kit insert, APTIMA HPV Assay, nr 503744En Rev. A). Samples with these HPV types, except HPV70 (low-risk for cancer), were also included in this study. HPV26 is a potential HR-type [15] as well as HPV67 [16]. In one study, HPV82 was classified as an HR-type [15]. Women were excluded if they did not have any subsequent histology sample registered within 4.5 years after the index cytology test.

Initially, 434 women with index ASCUS/LSIL and stored HR-HPV DNA-positive samples with subsequent histology were identified. The study was restricted to HR-HPV DNA-positive women only, since those are the women at risk of development of high grade CIN. We excluded samples that were positive for HPV types not covered by the APTIMA HPV assay (n = 21), samples that had been stored in SurePath medium for more than seven days prior to pelleting and freezing at -80°C (n = 60), and samples with low remaining volumes (less than 100 uL (n = 11)). In total, samples from 342 women were analysed; 211 with ASCUS, and 131 with LSIL at baseline.

### HPV E6/E7 mRNA

The samples were analysed in late 2013 to early 2014. According to APTIMA instructions (Jensen, D., Wilson, T., Kirkconell, B., Lee, E., George, P., Bennett, C., Weinbaum, B., Dockter, J.: Poster handout. 29th Clinical Virology Symposium, April 26-May 1, 2013, Daytona Beach, Florida), SurePath specimens were be pre-treated with proteinase K, included in the APTIMA Transfer Solution (ATS) kit. In the present study, 100 μL of the stored sample in STM was used, instead of 1 mL SurePath specimen. The used 100 μL corresponds to about 1/4 of the original 1 mL SurePath sample. Briefly, 100 μL of the sample and 300 μL of the ATS were added to an APTIMA Specimen Transfer Tube (pre-filled with 2.9 mL buffered saline solution) and heated at 90°C for 15 min in a water bath. The APTIMA HPV assay was performed according to the manufacturer's instructions using the PANTHER platform (Hologic).

### Statistical analysis

Analyses of sensitivity, specificity, positive predictive value and negative predictive value (including 95% confidence intervals) were calculated using a website for statistical computation (vassarstats.net). Fisher’s exact probability test (two-tailed) was used for analysis of distribution of high-grade lesions between HR-mRNA-positive and-negative women. P-values <0.05 were considered significant. Odds ratios (OR) were calculated using the LogExact software v.10.0.

## Results

The cohort of 342 HR-HPV-positive women between 35–89 years of age had cytological diagnoses of ASCUS or LSIL at baseline, and had histological follow-up of up to 4.5 years. Most women (61.4%) were between 35 and 44 years of age (Table 1).

**Table 1:** Number of positive and negative APTIMA HPV mRNA test results, and outcome after up to 4.5 years of follow-up.

			mRNA result (all samples)		CIN2+		CIN3	
Age	Index diagnosis	Total number	Positive	Negative	Positive	Negative	Positive	Negative
35–39	ASCUS	82	77	5	31	51	13	69
40–44	ASCUS	45	41	4	13	32	5	40
45–49	ASCUS	29	28	1	4	25	1	28
50–54	ASCUS	29	25	4	10	19	4	25
55–59	ASCUS	16	13	3	3	13	0	16
60–64	ASCUS	6	5	1	0	6	0	6
65–69	ASCUS	3	1	2	0	3	0	3
70–84	ASCUS	0	-	-	-	-	-	-
85–89	ASCUS	1	0	1	0	1	0	1
**35–89**	**Total:**	**211**	**190**	**21**	**61**	**150**	**23**	**188**
35–39	LSIL	43	41	2	15	28	6	37
40–44	LSIL	40	37	3	13	27	5	35
45–49	LSIL	21	21	0	8	13	4	17
50–54	LSIL	9	9	0	4	5	2	7
55–59	LSIL	7	7	0	2	5	2	5
60–64	LSIL	9	8	1	2	7	0	9
65–69	LSIL	2	2	0	1	1	1	1
70–89	LSIL	0	-	-	-	-	-	-
**35–89**	**Total:**	**131**	**125**	**6**	**45**	**86**	**20**	**111**

The HPV mRNA prevalence among the ASCUS and LSIL index samples was 90% (190/211; 95% CI 85.9–94.0) and 95.4% (125/131; 95% CI 91.8–99.0), respectively. After up to 4.5 years of follow-up of women with ASCUS or LSIL, 28.9% (61/211) and 34.3% (45/131), respectively, showed CIN2 or worse. The corresponding figures for CIN3 at follow-up were 10.9% (23/211) and 15.3% (20/131) (Table 2).

**Table 2:** Sensitivity, specificity, predictive value and odds ratio including 95% confidence interval for detecting CIN2+ (a) or CIN3 (b).

Initial diagnosis	**CIN2+**		**Sensitivity (95% CI)**	**Specificity (95% CI)**	**PPV (95% CI)**	**NPV (95% CI)**	**OR (95% CI)**
a)	Positive	Negative					
**ASCUS**							
*mRNA*							
Positive	59	131	96.7% (87.6–99.4)	12.7% (7.9–19.3)	31.0% (24.6–38.2)	90.5% (68.2–98.3)	4.3 (1.0–38.9)
Negative	2	19					
**CIN1**							
*mRNA*							
Positive	44	81	97.8% (86.8–99.9)	5.8% (2.2–13.6)	35.2% (27.0–44.3)	83% (36.5–99.1)	2.7 (0.3–131)
Negative	1	5					
	**CIN3**		**Sensitivity (95% CI)**	**Specificity (95% CI)**	**PPV (95% CI)**	**NPV (95% CI)**	**OR (95% CI)**
b)	Positive	Negative*					
**ASCUS**							
*mRNA*							
Positive	23	131	100% (82.2–100)	12.7% (8.0–19.3)	14.9% (9.9–21.8)	100% (79.1–100)	4.6 (0.7-INF)
Negative	0	19					
**CIN1**							
*mRNA*							
Positive	20	81	100% (80.0–100)	5.8% (2.2–13.6)	19.8% (12.8–29.2)	100% (46.3–100)	1.6 (0.2-INF)
Negative	0	5					

PPV, positive predictive value. NPV, negative predictive value. OR, odds ratio. INF, infinity.

* The reference group is women who did not develop CIN2+. Women who developed CIN2 are not included in this analysis.

The presence of HPV E6/E7 mRNA was associated with future development of CIN2+ among high-risk HPV DNA-positive women with ASCUS and LSIL (p = 0.02, OR; 3.9. 95% CI 1.1–20.5), and with future development of CIN3 (p = 0.03, OR; 6.8. 95% CI 1.1-INF).

Concerning mRNA, 90.0% (212/236) of non-progressors (e.g. not CIN2+ at follow up) were mRNA-positive at baseline (Table 2A). Among those who progressed to CIN2 or worse, 97.2% (103/106) manifested mRNA at baseline (Table 2A), and those with CIN3 at follow up showed 100.0% (43/43) mRNA positivity (Table 2B).

The APTIMA assay manifested a sensitivity of 96.7% for future CIN2+ and 100% for CIN3 among the ASCUS index group. For the LSIL group, the assay had a sensitivity of 97.8% for future CIN2+ and 100% of CIN3 (Table 2). The corresponding values of specificity, positive predictive value and negative predictive value are shown in Table 2.

Two women with ASCUS and one woman with LSIL developed CIN2 within 4.5 years, although the index samples were HPV mRNA-negative (Table 3).

**Table 3:** HPV types (DNA) detected in samples negative by the APTIMA HPV assay with a histologic diagnosis of CIN2.

Initial diagnosis	Age	Histologic follow-up diagnosis	HPV types detected
ASCUS	37	CIN2	30, 59, 81
ASCUS	36	CIN2	66
CIN1	37	CIN2	45, 53

No sample contained a single infection of HPV26. Two women were positive for HPV67 only and both expressed HPV mRNA; one of these women progressed to CIN2. Among six women positive for HPV82 only; three were mRNA-positive but none of the six women showed disease progression. Samples positive for HPV70 only (n = 1) were not included in the analysis.

## Discussion

We determined that the APTIMA HPV mRNA assay manifested high sensitivity in predicting future CIN2+ (96.7–97.8%) and CIN3 (100%) among HPV DNA-positive women with ASCUS or with LSIL at baseline, whereas the corresponding specificity was substantially lower (5.4–12.7%). Among these women with ASCUS or LSIL we also observed a high negative predictive value (100%) of the assay for development of future CIN3.

Slightly lower sensitivities compared to ours were reported by two Danish studies that also used the APTIMA mRNA test; 87.5% for CIN2+ and 92.6% for CIN3 among ASCUS index patients [17], and 92.5% for CIN2+ and 93.4% for CIN3 among LSIL index cytology patients [18]. A recent Swedish study with the same approach as ours reported a sensitivity for detecting future CIN2+ of only about 78.1% for ASCUS and LSIL index groups, and a sensitivity of 75.8% for development of CIN3 among those with LSIL at baseline [19]. However, for the ASCUS group the sensitivity for future CIN3 was 100%, which was in agreement with our result.

The low specificity of our study was due to the fact that 92.1% of all samples were HPV mRNA-positive at baseline. The Danish study, with 44.3% HPV DNA-positive ASCUS ThinPrep samples, showed a much higher specificity of 78.0% for detection of future CIN2+, and of 73.8% for future CIN3; compared to our study (CIN2+; 12.7% and CIN3; 12.7%) [17]. However, in their study of LSIL triage follow-up, the specificity was only 38.2% and 35.5% for future CIN2+ and CIN3, respectively [18]. In comparison to our study, the Swedish study also reported a higher specificity of 50.0% and 45.5% for future CIN2+ and CIN3 from ASCUS index patients, respectively [19]. They also reported higher specificities of 25.0% and 23.8% for future CIN2+ and CIN3 from LSIL index patients [19]. Their higher specificity might be due to that the APTIMA assay detected the remaining HPV mRNA in the long-time stored samples that initially were of high concentration [19].

In our study, all women that developed CIN3 within 4.5 years after the initial cytology diagnosis were positive for HPV-mRNA. Furthermore, our results showed that presence of HPV E6/E7 mRNA was associated with future development of CIN2+ among high-risk HPV DNA-positive women with ASCUS and LSIL (p = 0.02). However, lack of detectable HPV mRNA at baseline demonstrated a negative predictive value of 100% for CIN3, indicating that HPV-mRNA-negative women are at low risk for progression to high grade CIN. Thus, the outcome for our HPV mRNA-negative women are similar to large-scale studies that have shown that women who test negative for HPV-DNA have a very low risk of developing pre-cancer and cancer [20, 21].

Since three HPV-mRNA-negative women later developed CIN2, the negative predictive value for future CIN2+ was slightly lower (90.5% for ASCUS and 83.0% for LSIL). These three women had CIN2 diagnosed at 2–8 months after the initial diagnosis of ASCUS/LSIL, which harbored HPV-DNA for either HPV45, HPV59 or HPV66 (all covered by the APTIMA assay). The slightly lower HPV mRNA positivity at baseline for CIN2 cases might be due to overestimation at follow-up, since CIN2 diagnosis has shown relatively low reproducibility [22].

Our high negative predictive value for future CIN3 was not observed among LSIL samples in a recent Swedish study using a similar approach as ours [19]. Their APTIMA result of HPV DNA-positive LSIL cytology samples generated lower negative predictive values for future CIN2+ (78.1%) and CIN3 (75.8%). This means that the risk for future CIN3 remained for their women with LSIL index cytology even though the test was mRNA-negative. However, for the ASCUS index samples, their negative predictive values were comparable to ours.

It is important to note that of the women not progressing to CIN2+, 41.3% (62/150) of the ASCUS group and 54.6% (47/86) of the CIN1 group were treated by conization or hysterectomy within 4.5 years. Therefore, we cannot be sure that the treated women would have developed a more severe diagnosis if left untreated. Reasons for treatment of these women were repeated ASCUS or LSIL cytology. For some women conization was performed whereas others were hysterectomized because of problems with bleeding. These women were not excluded as they had a chance to develop a more serious lesion than ASCUS and LSIL. We do not suspect that the treatment had an impact on our results since the proportion of treated women were similar in HPV mRNA-positive and mRNA negative women. Among the mRNA-positive ASCUS and LSIL cases without progression to CIN2+, 41% (54/131) and 54% (44/81) were treated, respectively. Correspondingly, among the mRNA-negative ASCUS and LSIL cases 42% (8/19) and 60% (3/5) were treated, respectively.

The APTIMA HPV assay has only been validated for samples stored in PreservCyt medium (ThinPrep) (Hologic). However, we used a protocol for usage of fresh SurePath samples on the APTIMA HPV assay (Hologic). We analysed archival samples, originally collected in SurePath and stored between three and four years at -80°C. Based on our high prevalence of positive mRNA results (92.1%, positivity), we conclude that the mRNA in our archived SurePath samples is of good quality and is suitable for analysis. However, long-term storage of ThinPrep samples at room temperature might have negative effects on the quality and stability of the mRNA. For example, ThinPrep samples stored at room-temperature for five years showed an mRNA prevalence of 36% among HPV-DNA-positive samples [19]. Furthermore, the HPV mRNA prevalence of three-year-old archival ThinPrep LSIL samples was 67% [18]; however, the HPV DNA status of the samples was not investigated. There is no quality control of the sample mRNA in the APTIMA HPV assay. However, the assay includes an internal control for each test, which monitors the efficiency of the assay.

It is likely that some of the mRNA-negative women had low viral load of HPV, as described by Broccoli et al., [23]. The HPV mRNA-negative samples were collected in years 2009 and 2010, and were not biased for the oldest samples.

Other commercial assays such as the NucliSENS EasyQ HPV test (Biomerieux) [24] and PreTect HPV-Proofer, (Norchip) [25] detect oncogenic HPV E6/E7 mRNA of the five most common HR- HPV types (16, 18, 31, 33 and 45). Interestingly, in our study material, 53% (182/342) of the samples were HPV DNA-positive for one or more of these five HPV types.

In summary, we found the APTIMA HPV mRNA assay had high sensitivity but low specificity in detecting future high-grade CIN among HR-HPV DNA-positive women with ASCUS or LSIL cytology. Due to the high HPV mRNA positivity rates among the women without progressive disease, the specificity for predicting high grade CIN was low (5.8–12.7%). The presence of HPV E6/E7 mRNA was associated with future development of CIN2+ among high-risk HPV DNA-positive women with ASCUS and LSIL. The absence of HPV mRNA demonstrated a high negative predictive value for development of CIN3 among HPV-DNA-positive women with ASCUS or LSIL. Clear markers for progression are still needed to detect women at risk of development of high-grade lesions.
